# Potential of brain age in identifying early cognitive impairment in subcortical small-vessel disease patients

**DOI:** 10.3389/fnagi.2022.973054

**Published:** 2022-09-01

**Authors:** Yachen Shi, Haixia Mao, Qianqian Gao, Guangjun Xi, Siyuan Zeng, Lin Ma, Xiuping Zhang, Lei Li, Zhuoyi Wang, Wei Ji, Ping He, Yiping You, Kefei Chen, Junfei Shao, Xuqiang Mao, Xiangming Fang, Feng Wang

**Affiliations:** ^1^Department of Neurology, The Affiliated Wuxi People’s Hospital of Nanjing Medical University, Wuxi, China; ^2^Department of Interventional Neurology, The Affiliated Wuxi People’s Hospital of Nanjing Medical University, Wuxi, China; ^3^Department of Functional Neurology, The Affiliated Wuxi People’s Hospital of Nanjing Medical University, Wuxi, China; ^4^Department of Radiology, The Affiliated Wuxi People’s Hospital of Nanjing Medical University, Wuxi, China; ^5^Department of Neurosurgery, The Affiliated Wuxi People’s Hospital of Nanjing Medical University, Wuxi, China

**Keywords:** brain age, subcortical small-vessel disease, subcortical vascular cognitive impairment, gray matter volume, relevance vector regression

## Abstract

**Background:**

Reliable and individualized biomarkers are crucial for identifying early cognitive impairment in subcortical small-vessel disease (SSVD) patients. Personalized brain age prediction can effectively reflect cognitive impairment. Thus, the present study aimed to investigate the association of brain age with cognitive function in SSVD patients and assess the potential value of brain age in clinical assessment of SSVD.

**Materials and methods:**

A prediction model for brain age using the relevance vector regression algorithm was developed using 35 healthy controls. Subsequently, the prediction model was tested using 51 SSVD patients [24 subjective cognitive impairment (SCI) patients and 27 mild cognitive impairment (MCI) patients] to identify brain age-related imaging features. A support vector machine (SVM)-based classification model was constructed to differentiate MCI from SCI patients. The neurobiological basis of brain age-related imaging features was also investigated based on cognitive assessments and oxidative stress biomarkers.

**Results:**

The gray matter volume (GMV) imaging features accurately predicted brain age in individual patients with SSVD (*R*^2^ = 0.535, *p* < 0.001). The GMV features were primarily distributed across the subcortical system (e.g., thalamus) and dorsal attention network. SSVD patients with age acceleration showed significantly poorer Mini-Mental State Examination and Montreal Cognitive Assessment (MoCA) scores. The classification model based on GMV features could accurately distinguish MCI patients from SCI patients (area under the curve = 0.883). The classification outputs of the classification model exhibited significant associations with MoCA scores, Trail Making Tests A and B scores, Stroop Color and Word Test C scores, information processing speed total scores, and plasma levels of total antioxidant capacity in SSVD patients.

**Conclusion:**

Brain age can be accurately quantified using GMV imaging data and shows potential clinical value for identifying early cognitive impairment in SSVD patients.

## Introduction

Subcortical small-vessel disease (SSVD) is the most common cause of vascular cognitive impairment in elderly people and shows visible radiological biomarkers on magnetic resonance imaging (MRI), i.e., lacunar, white matter hyperintensity (WMH), cerebral microbleeds (CMBs), or brain atrophy (Chojdak-Łukasiewicz et al., 2021; Hamilton et al., 2021). Although the pathological mechanisms of SSVD remain unclear, there is strong evidence that potential pathophysiological mechanisms, including oxidative stress, inflammatory response, and endothelial dysfunction, can impair the structure and function of cerebral small vessels (Ungvari et al., 2021). Vascular dysfunction in SSVD can progressively induce neuronal damage and result in subcortical vascular cognitive impairment (SVCI), ranging in severity from subjective cognitive impairment (SCI) to mild cognitive impairment (MCI) and dementia (Gorelick et al., 2011; Diciotti et al., 2017). However, traditional imaging biomarkers are insufficiently accurate for identifying SVCI, especially early SVCI (Hosoki et al., 2021). Therefore, there is urgent need for new, more effective imaging biomarkers for SVCI, which will contribute to early interventions for cognitive impairment in SSVD patients.

Multimodal MRI has revealed abundant age-related changes in the brain microstructure of SSVD patients. Some subregions of the temporal lobes, hippocampus, and thalamus show decreased gray matter volume (GMV) and these changes are correlated with poor cognition in SSVD patients (Wang et al., 2020). In elderly people with WMH, higher functional connectivity in the fronto-parietal and salience networks may protect executive function (Benson et al., 2018). Patients with SSVD exhibit high heterogeneity in imaging characteristics; as a result, cognition-related imaging differences at the group-level are difficult for use as biomarkers at the individual level. Previous studies have indicated that brain age prediction can determine brain aging based on individual brain MRI data, which can be used as an effective biomarker to estimate cognitive impairment in neurological and psychiatric disorders (Liem et al., 2017; Shahab et al., 2019; Beheshti et al., 2020; Gautherot et al., 2021; Mishra et al., 2021). Thus, it is essential to investigate the association of SVCI with individual brain age.

Multivariate machine learning algorithms provide substantial advantages in processing multidimensional data and constructing personalized neuroimaging models (Shi et al., 2021b; Wang et al., 2021). Although some algorithms have been used to analyze cerebral WMH and diffusion tensor imaging data, machine learning in SSVD is preliminary (Ciulli et al., 2016; Zee et al., 2021). To date, no study has explored the potential of machine learning for the analysis of multimodal MRI data in SSVD. Among the available machine learning methods, the relevance vector regression (RVR) algorithm offers great advantages for reducing model complexity and increasing predictive efficacy and has been widely used to build effective prediction models, including brain age prediction models (Cui and Gong, 2018; Hajek et al., 2019; Baecker et al., 2021).

**FIGURE 1:**
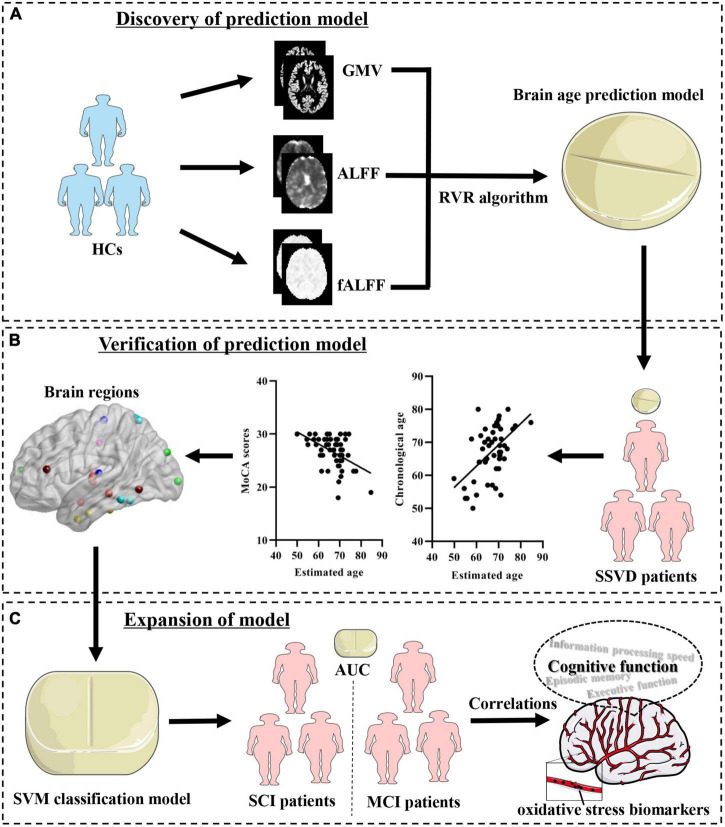
Schematic of the data analysis pipeline. **(A)** Discovery of prediction model. Imaging feature extraction for each brain region was based on the Brainnetome atlas and used to establish brain age prediction models. **(B)** Verification of the prediction model. Determination of the brain age prediction model in SSVD patients and evaluation of the association of brain age with cognitive assessments. **(C)** Expansion of the model. Establishment of a SVM classification model with brain age-related imaging features and exploration of its neurobiological basis. HCs, healthy controls; SSVD, subcortical small-vessel disease; GMV, gray matter volume; mALFF, mean amplitude of low frequency fluctuation; mfALFF, mean fractional amplitude of low-frequency fluctuation; RVR, relevance vector regression; SVM, support vector machine; SCI, subjective cognitive impairment; MCI, mild cognitive impairment; AUC, area under the curve.

The aim of the present study is to build a brain age predication model and evaluate the relationship between brain age and cognitive impairments in SSVD. Brain age-related neuroimaging features are then identified and their potential clinical applications in SVCI are further explored. The study design and analyses are shown in Figure 1.

## Materials and methods

### Participants

Thirty-five healthy controls (HCs) were recruited through community health screening and 51 SSVD patients were recruited from The Affiliated Wuxi People’s Hospital of Nanjing Medical University (Wuxi, China). All participants underwent a standardized clinical interview, including demographic inventory and examination of their physical and mental health. Routine blood testing and brain imaging (three-dimensional T1-weighted, T2-weighted, fluid-attenuated inversion recovery, susceptibility weighted images, diffusion-weighted imaging, and magnetic resonance angiography) were performed for each participant. All participants or their legal guardians provided written informed consent after being explained all details of the study. The ethical approval was obtained from the Ethics Committee of the Affiliated Wuxi People’s Hospital of Nanjing Medical University (approval number: KY2112).

### Neuropsychological assessments

All participants underwent a neuropsychological battery comprising the following tests: (1) global cognition: Mini-Mental State Examination (MMSE) and Montreal Cognitive Assessment (MoCA); (2) episodic memory: Auditory Verbal Learning Test-immediate recall (AVLT-IR) and Auditory Verbal Learning Test-20-min delayed recall (AVLT-20 min DR); (3) information processing speed: Trail Making Tests A (TMT-A) and Stroop Color and Word Test A and B (Stroop-A and Stroop-B); (4) executive function: Trail Making Tests B (TMT-B), Stroop Color and Word Test C (Stroop-C), and Digit Span Test (DST); (5) visuospatial function: Clock Drawing Test (CDT); (6) emotional assessment: 17-item Hamilton Depression Rating Scale (HAMD-17), and (7) National Institutes of Health Stroke Scale (NIHSS) was also conducted.

To facilitate data analysis, standard data translation was performed as previously described (Shu et al., 2016; Shi et al., 2020a). The raw test scores underwent Z-transformation using the mean and standard deviation. To obtain the total information processing speed and executive function scores, the composite scores for these two cognitive domains were calculated using the Z-transformed averages.

### Inclusion and exclusion criteria

The general inclusion criteria for all participants were as follows: (1) aged 50−80 years old; (2) ≥ 6 years of education; (3) generally in good health with adequate visual and auditory acuity for neuropsychological assessments; and (4) no contraindication to MRI scanning. All HCs showed normal cognition (MMSE score ≥ 26, MoCA score ≥ 26, and HAMD-17 < 7) without any symptoms of stroke or imaging changes reflecting cerebrovascular disease.

According to established diagnostic criteria (Pantoni, 2010; Chen et al., 2019), SSVD patients were diagnosed based on MRI evidence of vascular changes as follows: (1) total number of lacunes were counted, and ≥ 2 lacunes were considered as the presence of lacunes; (2) WMH was quantified using the Fazekas scale (overall score of 6), and a score ≥ 1 was considered as displaying WMH; and (3) the total number of CMBs were counted, and ≥ 3 CMBs was considered as a threshold. Among the SSVD patients, those with MCI exhibited some degree of cognitive impairment as confirmed by family members and neuropsychological assessments, without meeting the Diagnostic and Statistical Manual of Mental Disorders, Fifth Edition criteria for dementia. SSVD patients with SCI had subjective complaints about impaired cognitive function, but there was a lack of objective evidence of cognitive impairment (i.e., normal neuropsychological assessments).

Participants with any of following characteristics were excluded: (1) major depressive disorder or other psychiatric disorders; (2) clinical cerebrovascular disorders with large intracranial vascular lesion; (3) a history of brain trauma or other neurologic disease (e.g., Parkinson’s disease); (4) significant medical problems (e.g., autoimmune disease, tumor, significantly impaired liver, or kidney function); and (5) abuse or dependence of alcohol or drugs.

### Magnetic resonance imaging data acquisition and preprocessing

Multimodal imaging data acquisition and preprocessing have been described in our previous studies (He et al., 2019; Shi et al., 2019; Wang et al., 2021) and Supplementary material. A 3.0T MR scanner (MAGNETOM Prisma, Siemens Healthcare, Germany) was used in the present study. The average GMV values of 210 cortical and 36 subcortical subregions (Supplementary Table 1), as described in the Brainnetome Atlas (Fan et al., 2016), were obtained for each participant. Processing of the mean amplitude of low frequency fluctuation (mALFF) and mean fractional amplitude of low-frequency fluctuation (mfALFF) are described in Supplementary material. The values of the MRI indicators were utilized as feature vectors for the subsequent analyses.

### Multivariate relevance vector regression analysis

Multivariate RVR analysis was described in our previous studies (Shi et al., 2020b,2021a) and additional details of RVR method (Tipping, 2001) are provided in the Supplementary material. In brief, the association between age and GMV values was analyzed using a multivariate RVR method implemented in the Pattern Recognition for Neuroimaging Toolbox.^1^ In the training and test sets, leave-one-out cross-validation (LOOCV) was performed to evaluate the generalizability of the model (Cui and Gong, 2018). The Pearson correlation coefficient (R) and mean absolute error (MAE) between chronological age and estimated age were used to evaluate the prediction performance of the model (Feng et al., 2019). Next, a permutation test was performed to determine the significance of r and MAE. The above-mentioned processing method was also performed for the single mALFF, single mfALFF features, and combined GMV and mALFF/mfALFF features (Supplementary Table 2).

In the present study, HCs were used as the training set to determine the optimal prediction model. Subsequently, the prediction model was utilized for SSVD patients (as the test set) to predict individual brain age. The weight of each imaging feature can quantify its contribution in the prediction model; an imaging feature was retained if the absolute value of its weight was in the top 10%, thereby retaining the most predictive imaging features. Furthermore, to better annotate the distribution of imaging features, eight brain networks including the seven large-scale functional modules and one subcortical module, were assigned 246 brain regions (Yeo et al., 2011). These brain networks included visual network, sensorimotor network, dorsal attention network, ventral attention network, limbic network, fronto-parietal network, default mode network, and subcortical system.

### Support vector machine classification

The LIBSVM toolbox of MATLAB was used to construct the SVM classification model (Hsu and Lin, 2002) to distinguish SSVD patients with MCI from SCI ones. LOOCV was used to assess the generalizability of the model while the average accuracy, sensitivity, and specificity were used to evaluate its performance. The area under the curve (AUC) of the receiver operating characteristic curve was used to determine the classification capacity of the model.

### Measurement of oxidative stress biomarkers

Peripheral venous blood was collected from each SSVD patient between 8:00-9:00 a.m. after overnight fasting in EDTA-coated tubes. Within 30 min of collection, samples were centrifuged at 1000 *g* for 10 min at 4°C to obtain plasma. The plasma sample was aliquoted and stored at −80°C.

Three common antioxidant indexes were measured: the levels of superoxide dismutase (SOD), catalase (CAT), and total antioxidant capacity (T-AOC) in plasma were determined using a fully automatic biochemical analyzer (Beckman Coulter UniCel DxC 600 Synchron, United States). Human plasma samples were assayed using SOD assay kits (Lot: 20220712.40001, RGB, Beijing, China), CAT assay kits (Lot: 20221113.40051, RGB, Beijing, China), and T-AOT assay kits (Lot: 20220715.40009, RGB, Beijing, China).

### Sample size calculation

The sample size was determined using an online sample size calculator.^2^ The sample size for the SCI and MCI groups in this study were roughly based on the results using α = 0.05 and β = 0.2. Additional details can be found in the Supplementary material and Supplementary Table 2.

### Statistical analysis

SPSS version 20.0 software (IBM Corp., Armonk, NY, United States) was used for the present statistical analyses. Continuous variables were analyzed using the independent-samples *t*-test in cases with a normal distribution as determined using the Kolmogorov–Smirnov test, while the Mann–Whitney U test was used in cases showing a non-normal distribution. Bonferroni correction was used for multiple comparison using one-way ANOVA, and Nemenyi correction was used for multiple comparison using Kruskal–Wallis. Categorical variables were analyzed using the Chi-square test. Pearson correlation analysis and partial correlation analysis (controlling for chronological age, sex, years of education, and NIHSS score) were used to determine the relationship between estimated age and cognitive function as well as the relationship between the classification outputs and cognitive assessment scores and plasma levels of oxidative stress biomarkers in SSVD patients. Results with *p* < 0.05 were considered to be statistically significant.

## Results

### Participant characteristics

The clinical features of the 35 HCs and 51 SSVD patients are shown in Table 1. Data are also provided for the SSVD patient subgroups, namely the SCI group (*n* = 24) and MCI group (*n* = 27). There were significant differences in sex, cognitive assessment scores (i.e., MMSE, MoCA, AVLT-IR, AVLT-20 min DR, TMT-A, TMT-B, Stroop-B, Stroop-C, information processing speed, and executive function), and plasma T-AOC levels between the SCI and MCI groups (Table 1). In addition, among HC, SCI, and MCI groups, MMSE, MoCA, AVLT-IR, AVLT-20 min DR, TMT-A, TMT-B, Stroop-B, Stroop-C, information processing speed, and executive function scores were also significantly difference (Supplementary Figure 1).

**TABLE 1:** Demographic information, cognitive assessment scores, and plasma data for HC and SSVD participants.

	HC (*n* = 35)	SSVD (*n* = 51)		
Age (years)	60.57 ± 7.80	67.16 ± 7.80		
Sex (Male/Female)	20/15	22/29		
Education (years)	11.54 ± 3.42	9.80 ± 2.66		
Fazekas score	0.00 ± 0.00	2.08 ± 1.25		
NIHSS scores	0.00 ± 0.00	0.18 ± 0.48		
MMSE scores	28.86 ± 1.33	27.98 ± 1.79		
MoCA	28.71 ± 1.67	26.45 ± 3.49		
HAMD-17 scores	1.23 ± 2.30	1.76 ± 2.30		

		**SCI (*n* = 24)**	**MCI (*n* = 27)**	***P-value***

Age (years)		65.29 ± 7.74	68.81 ± 7.62	0.108*
Sex (Male/female)		3/21	19/8	< 0.001^&^
Education (years)		9.96 ± 2.84	9.67 ± 2.54	0.700*
Fazekas score		1.833 ± 1.24	2.30 ± 1.23	0.134^#^
NIHSS score		0.17 ± 0.48	0.10 ± 0.30	0.561*
MMSE score		29.00 ± 1.06	27.22 ± 1.69	< 0.001*
MoCA score		28.42 ± 1.25	25.04 ± 3.12	< 0.001*
HAMD-17 scores		1.58 ± 2.00	1.78 ± 2.56	0.766*
AVLT-IR (raw score)		7.19 ± 1.94	4.95 ± 1.91	< 0.001*
AVLT-IR (Z score)		0.54 ± 0.87	−0.48 ± 0.86	< 0.001*
AVLT-20 min DR (raw score)		6.67 ± 2.91	4.07 ± 2.67	0.002*
AVLT-20 min DR (Z score)		0.45 ± 0.95	−0.40 ± 0.88	0.002*
TMT-A (raw score)		56.58 ± 16.38	80.67 ± 19.13	< 0.001*
TMT-A (Z score)		−0.59 ± 0.76	0.53 ± 0.89	< 0.001*
Stroop-A (raw score)		28.21 ± 6.85	31.26 ± 5.95	0.095*
Stroop-A (Z score)		−0.25 ± 1.05	0.22 ± 0.91	0.095*
Stroop-B (raw score)		42.88 ± 11.37	61.67 ± 14.72	< 0.001*
Stroop-B (Z score)		−0.61 ± 0.70	0.55 ± 0.91	< 0.001*
Information processing speed		−0.49 ± 0.66	0.43 ± 0.64	< 0.001*
TMT-B (raw score)		137.88 ± 25.48	271.93 ± 168.64	< 0.001^#^
TMT-B (Z score)		−0.51 ± 0.18	0.45 ± 1.20	< 0.001^#^
Stroop-C (raw score)		77.63 ± 19.30	142.41 ± 52.79	< 0.001*
Stroop-C (Z score)		−0.66 ± 0.37	0.59 ± 1.02	< 0.001*
DST-backward (raw score)		4.54 ± 0.72	4.22 ± 0.64	0.053^#^
DST-backward (Z score)		0.24 ± 1.04	−0.22 ± 0.93	0.053^#^
Executive function		−0.31 ± 0.31	0.27 ± 0.71	0.001*
CDT (raw score)		8.54 ± 0.98	8.22 ± 1.05	0.360^#^
CDT (Z score)		0.17 ± 0.96	−0.15 ± 1.03	0.360^#^
Plasma SOD (U/ml)		106.22 ± 12.20	103.80 ± 7.46	0.227*
Plasma CAT (U/ml)		6.58 ± 2.77	5.92 ± 2.36	0.367*
Plasma T-AOC (U/ml)		11.80 ± 3.31	9.31 ± 2.28	0.003*

(1) Data are presented as the mean ± standard deviation. (2) The SCI and MCI groups were two sub-groups of the SSVD group. (3) The information processing speed total scores were calculated using the TMT-A, Stroop-A, and Stroop-B scales (Z scores) scores, while the executive function total scores were calculated using the TMT-B, Stroop-C, and DST-backward scale (Z scores) scores. HC, healthy control; SSVD, subcortical small-vessel disease; SCI, subjective cognitive impairment; MCI, mild cognitive impairment; NIHSS, National Institutes of Health Stroke Scale; MMSE, Mini-mental State Examination; MoCA, Montreal Cognitive Assessment; AVLT-IR, Auditory Verbal Learning Test-immediate recall; AVLT-20 min DR, Auditory Verbal Learning Test-20-min delayed recall; TMT-A, Trail Making Test A; Stroop-A, Stroop Color and Word Test A; Stroop-B, Stroop Color and Word Test B; TMT-B, Trail Making Test B; Stroop-C, Stroop Color and Word Test C; DST, Digit Span Test; CDT, Clock Drawing Test; SOD, superoxide dismutase; CAT, catalase; T-AOC, total antioxidant capacity.

*P-values were obtained by Independent-Samples T-test.

^#^P-values were obtained by Mann-Whitney U test.

^&^P-values were obtained by Chi-square test.

### Discovery of prediction model

**FIGURE 2:**
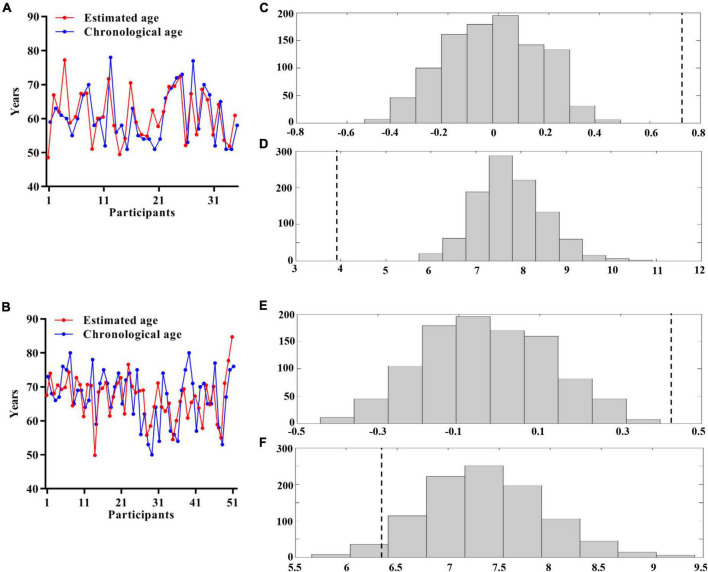
Multivariate relevance vector regression analysis. Scatterplot showing the estimated age for each participant derived from their imaging features compared with their chronological age (**A:** HCs; **B:** SSVD patients). Distribution of permutation of the prediction R and mean absolute error (**C,D:** HCs; **E,F:** SSVD patients). The values obtained using real scores are indicated by the dashed line. HCs, healthy controls; SSVD, subcortical small-vessel disease.

To develop an optimal prediction model, 35 HCs with MRI features were used as the training set. Application of the RVR algorithm to the GMV features (*R*^2^ = 0.535, *p* < 0.001, MAE = 3.916, *p* < 0.001; Figures 2A,C,D) achieved better individualized prediction of brain age than other MRI features (Supplementary Table 3). Therefore, the RVR prediction model with GMV features was retained.

### Prediction of brain age in subcortical small-vessel disease patients

The optimal prediction model with GMV features, determined from the training set, was then tested in the 51 SSVD patients. Figure 2B shows that the application of the RVR algorithm to the GMV data allowed quantitative prediction of each patient’s brain age with statistically significant accuracy (*R*^2^ = 0.183, *p* < 0.001, MAE = 6.425, *p* = 0.045; Figures 2B,E,F).

**FIGURE 3:**
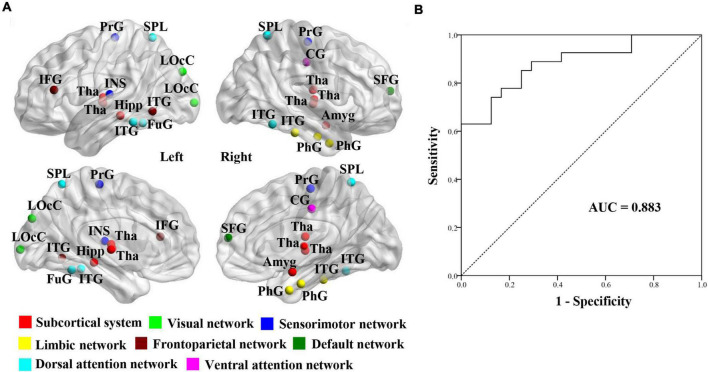
Establishment of SVM classification model. **(A)** Visualizations of 25 gray matter volume features using relevance vector regression analysis for the prediction of brain age in SSVD patients. **(B)** Classification Performance of SVM classification model between SCI patients and MCI patients in participants with SSVD. SSVD, subcortical small-vessel disease; SVM, support vector machine; AUC, area under the curve.

The 25 brain regions that contributed most to RVR prediction were identified by setting the threshold to ≥ 10% of the maximum weight vector value (Table 2). These regions were mainly localized in the subcortical system (especially the thalamus) and dorsal attention network (Figure 3A).

**TABLE 2:** Contributing GMV features and their weight scores in the RVR algorithm used to predict brain age in SSVD patients.

Neuroanatomical region	Weight score	MNI (x, y, z)
SFG, medial area 10 (R)	0.1097	8, 58, 13
IFG, inferior frontal sulcus (L)	0.1093	−47, 32, 14
PrG, area 4 (upper limb region) (L)	0.1119	−26, −25, 63
PrG, area 4 (upper limb region) (R)	0.1060	34, −19, 59
ITG, extreme lateroventral area 37 (R)	0.2470	53, −52, −18
ITG, caudolateral of area 20 (L)	0.1608	−59, −42, −16
ITG, ventrolateral area 37 (L)	0.1597	−55, −60, −6
ITG, caudoventral of area 20 (R)	0.1508	54, −31, −26
FuG, lateroventral area 37 (L)	0.1429	−42, −51, −17
PhG, area 28/34 (EC, entorhinal cortex) (R)	0.0985	19, −10, −30
PhG, area TI (temporal agranular insular cortex) (R)	0.1482	22, 1, −36
SPL, rostral area 7 (L)	0.2543	−16, −60, 63
SPL, rostral area 7 (R)	0.1115	19, −57, 65
INS, hypergranular insula (L)	0.1002	−36, −20, 10
CG, caudal area 23 (R)	0.1077	6, −20, 40
LOcC, occipital polar cortex (L)	0.1056	−18, −99, 2
LOcC, medial superior occipital gyrus (L)	0.1265	−11, −88, 31
Amyg, medial amygdala (R)	0.1456	19, −2, −19
Hipp, caudal hippocampus (L)	0.1075	−28, −30, −10
Tha, pre-motor thalamus (L)	0.2542	−18, −13, 3
Tha, pre-motor thalamus (R)	0.1275	12, −14, 1
Tha, rostral temporal thalamus (L)	0.1969	−7, −14, 7
Tha, rostral temporal thalamus (R)	0.1572	3, −13, 5
Tha, caudal temporal thalamus (R)	0.1232	10, −14, 14
Tha, lateral pre-frontal thalamus (L)	0.1558	−11, −14, 2

GMV, gray matter volume; RVR, relevance vector regression; SSVD, subcortical small-vessel disease; SFG, superior frontal gyrus; IFG, inferior frontal gyrus; PrG, precentral gyrus; ITG, inferior temporal gyrus; FuG, fusiform gyrus; PhG, parahippocampal gyrus; SPL, superior parietal lobule; SPL, superior parietal lobule; INS, insular gyrus; CG, cingulate gyrus; LOcC, lateral occipital cortex; Amyg, amygdala; Hipp, hippocampus; Tha, thalamus.

Among SSVD patients, the estimated ages were significantly correlated with MoCA, AVLT-IR, TMT-A, TMT-B, and Stroop-C scores, and information processing speed and executive function total scores (Table 3). Moreover, these correlations remained after controlling for chronological age, sex, years of education, and NIHSS score (Table 3). However, chronological age of SSVD patients showed less association with cognitive assessment scores (Table 3). Furthermore, further correlation analyses in MCI, SCI, and HC groups, respectively (Supplementary Tables 4–6), indicated that the estimated ages of MCI patients showed stronger correlation with executive function than SCI patients, and abundant associations of chronological age with cognitive assessments could be found in the health status rather than the disease state.

**TABLE 3:** Association of age with cognitive function and plasma antioxidant index levels in SSVD patients.

	chronological age*	estimated age*	estimated age^#^	brain age gap*	brain age gap^#^
MMSE	*r* = 0.129; *p* = 0.367	***r* = −0.324; *p* = 0.020**	***r* = −0.336; *p* = 0.021**	***r* = −0.410; *p* = 0.003**	***r* = −0.383; *p* = 0.008**
MoCA	*r* = 0.103; *p* = 0.474	***r* = −0.408; *p* = 0.003**	***r* = −0.422; *p* = 0.003**	***r* = −0.424; *p* = 0.002**	***r* = −0.418; *p* = 0.003**
AVLT-IR	*r* = −0.122; *p* = 0.392	***r* = −0.288; *p* = 0.041**	***r* = −0.296; *p* = 0.043**	*r* = −0.215; *p* = 0.129	***r* = −0.334; *p* = 0.022**
AVLT-20 min DR	*r* = 0.022; *p* = 0.881	*r* = −0.250; *p* = 0.077	*r* = −0.264; *p* = 0.073	*r* = −0.253; *p* = 0.073	*r* = −0.278; *p* = 0.058
TMT-A	***r* = 0.356; *p* = 0.010**	***r* = 0.455; *p* = 0.001**	***r* = 0.420; *p* = 0.003**	*r* = 0.188; *p* = 0.188	***r* = 0.429; *p* = 0.003**
TMT-B	*r* = 0.140; *p* = 0.326	***r* = 0.441; *p* = 0.001**	***r* = 0.403; *p* = 0.005**	***r* = 0.388; *p* = 0.005**	***r* = 0.508; *p* < 0.001**
Stroop-A	*r* = 0.045; *p* = 0.754	*r* = 0.189; *p* = 0.183	*r* = 0.199; *p* = 0.180	*r* = 0.170; *p* = 0.234	*r* = 0.222; *p* = 0.133
Stroop-B	*r* = 0.066; *p* = 0.646	*r* = 0.183; *p* = 0.198	*r* = 0.169; *p* = 0.255	*r* = 0.118; *p* = 0.409	*r* = 0.166; *p* = 0.266
Stroop-C	*r* = 0.211; *p* = 0.137	***r* = 0.400; *p* = 0.004**	***r* = 0.375; *p* = 0.009**	*r* = 0.274; *p* = 0.052	***r* = 0.438; *p* = 0.002**
DST-backward	*r* = −0.100; *p* = 0.485	*r* = −0.025; *p* = 0.862	*r* = −0.029; *p* = 0.846	*r* = 0.021; *p* = 0.885	*r* = −0.021; *p* = 0.891
CDT	*r* = −0.055; *p* = 0.700	*r* = −0.269; *p* = 0.056	*r* = −0.269; *p* = 0.068	*r* = −0.216; *p* = 0.128	*r* = −0.273; *p* = 0.064
Information processing speed	*r* = 0.197; *p* = 0.166	***r* = 0.349; *p* = 0.012**	***r* = 0.334; *p* = 0.022**	*r* = 0.200; *p* = 0.159	***r* = 0.347; *p* = 0.017**
Executive function	*r* = 0.113; *p* = 0.429	***r* = 0.359; *p* = 0.010**	***r* = −0.322; *p* = 0.027**	***r* = 0.313; *p* = 0.025**	***r* = 0.399; *p* = 0.005**
Plasma SOD (U/ml)	*r* = −0.199; *p* = 0.162	*r* = 0.105; *p* = 0.464	*r* = 0.153; *p* = 0.306	*r* = 0.207; *p* = 0.144	*r* = 0.135; *p* = 0.365
Plasma CAT (U/ml)	*r* = 0.122; *p* = 0.395	*r* = 0.074; *p* = 0.606	*r* = 0.072; *p* = 0.629	*r* = −0.022; *p* = 0.879	*r* = 0.052; *p* = 0.731
Plasma T-AOC (U/ml)	*r* = −0.260; *p* = 0.065	*r* = −0.220; *p* = 0.120	*r* = −0.126; *p* = 0.398	*r* = −0.040; *p* = 0.783	*r* = −0.151; *p* = 0.311

(1) Z scores of other assessments were used for the present analysis except for the use of raw scores of the MMSE and MoCA. (2) The information processing speed total scores were calculated using the TMT-A, Stroop-A, and Stroop-B scales (Z scores) scores, while the executive function total scores were calculated using the TMT-B, Stroop-C, and DST-backward scale (Z scores) scores. (3) Brain age gap = (estimated age - chronological age). SSVD, subcortical small-vessel disease; MMSE, Mini-mental State Examination; MoCA, Montreal Cognitive Assessment; AVLT-IR, Auditory Verbal Learning Test-immediate recall; AVLT-20 min DR, Auditory Verbal Learning Test-20-min delayed recall; TMT-A, Trail Making Test-A; Stroop, Stroop Color and Word Test; TMT-B, Trail Making Test-B; DST, Digit Span Test; CDT, Clock Drawing Test; SOD, superoxide dismutase; CAT, catalase; T-AOC, total antioxidant capacity.

*P-values were obtained by Pearson correlation test.

^#^P-values were obtained by Partial correlation test (adjusting chronological age, sex, years of education, and NIHSS score).

Bold represents statistically significant.

Next, the brain age gap (estimated age − chronological age) was calculated for each SSVD patient. Regardless of whether chronological age, sex, education years, and NIHSS score were controlled, the brain age gap was negatively correlated with MMSE and MoCA scores and positively correlated with TMT-B scores and executive function total scores in SSVD patients (Table 3). Meanwhile, more associations between the brain age gap and cognitive assessments were observed in the MCI group than the SCI group, and no association were found in the HC group (Supplementary Tables 4–6).

Additionally, age acceleration (AgeAccel) (Monseur et al., 2020) is defined as estimated age > 2 years older than chronologic age, which will avoid the number in the AgeAccel group thins quickly (e.g., 14 and 6 cases of AgeAccel if using > 5 and > 10, respectively). As a result, there were 21 SSVD patients with AgeAccel and 31 ones without AgeAccel in the present study. Significantly, SSVD patients with AgeAccel were noted to have lower MMSE scores [27.45 vs. 28.45 (*p* = 0.036)] and lower MoCA scores [25.40 vs. 27.41 (*p* = 0.015)] than those without AgeAccel.

### Building a support vector machine model in subcortical small-vessel disease patients

To determine whether brain age-related GMV features can contribute to distinguishing SSVD patients with MCI from ones with SCI, a SVM classification model was built using the 25 GMV features derived from the RVR prediction model (Figure 3A). The classification accuracy of the SVM model was 80.4% (sensitivity = 70.8%, specificity = 88.9%) and the AUC value was 0.883 (Figure 3B).

### Relationships between support vector machine model decision values and cognitive ability and plasma oxidative stress indicator levels

**FIGURE 4:**
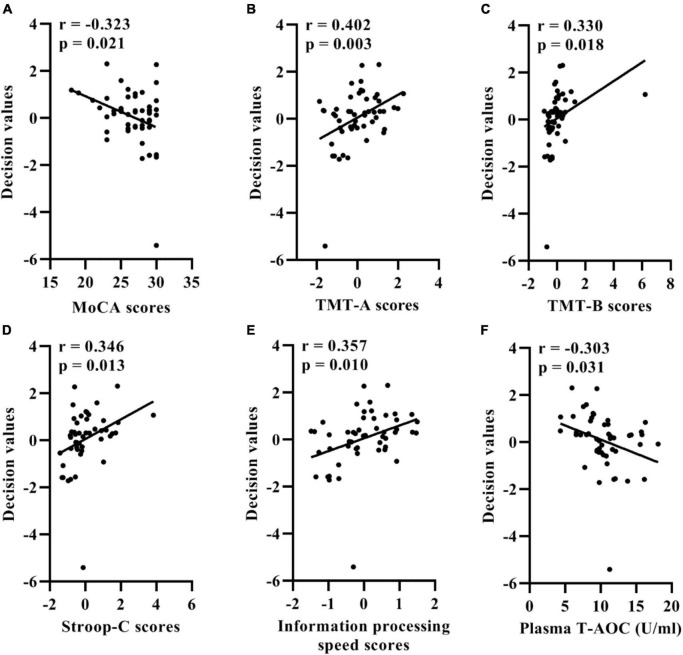
Correlation between the support vector machine model classification probabilities and MoCA **(A)**, TMT-A **(B)**, TMT-B **(C)**, Stroop-C **(D)** scores, information processing speed total scores **(E)**, and plasma levels of T-AOC **(F)** in patients with SSVD. SSVD, subcortical small-vessel disease; MoCA, Montreal Cognitive Assessment; TMT-A, Trail Making Test A; TMT-B, Trail Making Test B; Stroop-C, Stroop Color and Word Test C; T-AOC, total antioxidant capacity.

Among SSVD patients, the MoCA, TMT-A, TMT-B, and Stroop-C scores were significantly correlated with the classification outputs (Figures 4A−D). In addition, the information processing speed total scores and classification outputs showed significant associations (Figure 4E). A significant correlation of the classification outputs with the plasma levels of T-AOC was detected in SCI and MCI patients (Figure 4F). Controlling for chronological age, sex, years of education, and NIHSS score, the association of MoCA and TMT-A scores with the classification outputs was significant statistically in SSVD patients (MoCA: correlation coefficient = −0.327, *p* = 0.025; TMT-A: correlation coefficient = 0.323, *p* = 0.027).

## Discussion

In the present study, individualized brain age was predicted for HCs using the RVR algorithm. Subsequently, the reproducibility and generalizability of the RVR model was determined in SSVD patients and the distinguishing power of brain age-related imaging features were systematically investigated in SSVD patients with SCI and MCI. We found that the GMV features could predict brain age of individual HCs and patients with SSVD with great accuracy. The SVM classifier developed with 25 brain age-related GMV features could distinguish between SCI and MCI patients with an AUC of 0.883. Importantly, the estimated age of SSVD patients showed better correlation with current cognitive function than chronological age, especially for MCI patients, and the classification outputs also showed significant associations with the various cognitive assessment measures and antioxidant index levels. Taken together, these findings indicate that GMV MRI biomarkers can predict individual brain aging and contribute to identifying SSVD patients with a high risk of cognitive impairment.

Consistent with previous studies (Cole, 2020; Demro et al., 2022), structural MRI features showed prominent potential for the prediction of brain age. In the present study, brain age-related GMV features were mainly distributed in the subcortical system (e.g., thalamus and hippocampus) and dorsal attention network. Autopsy findings have indicated a potential link between an impaired thalamus and severe cognitive impairment in SSVD patients (Gorelick et al., 2011). Thong et al. (2014) reported that SVCI patients exhibited significantly decreased GMV in the thalamus compared to normal controls. Furthermore, SSVD patients showed abnormal function connectivity between the dorsal attention network and other networks (Liu et al., 2019) and, compared with HCs, significant GMV reductions were found in brain regions corresponding to the dorsal attention network in subjects with cognitive impairment (Joshi et al., 2019). Thus, simultaneous gray matter atrophy of multiple brain regions appears to be an important basis of brain aging and impaired cognitive function in SSVD patients.

In the present study, the predicted brain age of SSVD patients exhibited a strong association with cognitive decline. Compared with chronological age, the estimated age based on objective imaging features showed significant associations with global cognitive function and executive function scores, which suggests that individual brain age can reflect cognitive function in SVCI. Furthermore, by calculating the brain age gap, we observed a pattern such that SSVD patients with worse cognitive function had an older estimated age, which is similar to studies of Alzheimer’s disease (Gaser et al., 2013; Cheng et al., 2021). Meanwhile, further analyses indicated that significantly, SSVD patients with AgeAccel had lower MMSE and MoCA scores. Altogether, brain age is a potential biomarker of cognitive impairment in SSVD patients, which represents an accelerated aging in some brain structures involving in cognition.

To explore the underlying mechanism of brain age in SVCI, brain age-related MRI features were further investigated. Using 25 brain age-related GMV features, a SVM-based classification model was constructed and shown to accurately classify SSVD patients with MCI from those with SCI at the individual level. The present study also found significant correlations between the classification outputs of SVM model and cognitive assessment scores, indicating that the probability of identifying SVCI patients depended on the degree of cognitive decline. A previous study showed that a worse cognitive capacity was significantly associated with lower plasma T-AOC levels in older adults (Alghadir et al., 2021). Our results showed that the classification outputs were related to plasma levels of T-AOC, which suggests that the diagnostic model for SVCI patients performs better with poor antioxidative stress levels. These findings illustrate a potential association between brain age-related MRI features and the personalized clinical features of SVCI.

In the present study, individual brain age was estimated accurately and conveniently in SSVD patients using objective MRI biomarkers for the first time. We used a strict training set and test set (two independent cohorts) to construct the RVR prediction model to assure its reproducibility and generalizability. In addition, important brain age-related brain regions were determined and their clinical value in SVCI was further assessed *via* clinical manifestations and molecular changes.

Some limitations of the present study should be noted. First, the study included a small sample of SCI and MCI patients. Although a sample size calculation was performed to ensure an acceptable sample size (Supplementary Table 2), a larger sample of SVCI patients is necessary for testing the present SVM classification model and dementia patients should be included in a subsequent study. Moreover, a balanced distribution of sex in SSVD patients should be also considered in the future. Second, although the combined GMV and mALFF/mfALFF features showed a non-ideal performance to predict brain age compared to single GMV features in the present study, additional MRI features (e.g., functional regional homogeneity, functional connectivity, cortical thickness, etc.,) should be considered for achieving data fusion in future studies. Meanwhile, abundant biomarkers (e.g., whole-blood, cerebrospinal fluid) and many potential algorithms in search of capturing accurate and reliable biomarkers are valuable approaches to improve the prediction accuracy of brain age. Third, there was significant differences in mean age between HC and SSVD group although they had the obvious overlap in age distribution (HC: minimum value = 51, maximum value = 78; SSVD: minimum value = 50, maximum value = 80). In the present study, RVR prediction model showed better generalizability and predictive performance for each participant, however, same age range between training and test sets may further improve the performance of model. Lastly, additional cardiovascular risk factors [e.g., genetic information, high blood pressure, alcohol intake (de Lange et al., 2020)] should be incorporated to evaluate the clinical value of brain age in SVCI.

## Conclusion

Gray matter volume imaging data can be used to quantitatively and accurately predict brain age in individual patients with SSVD based on a multivariate RVR algorithm. This model provides an important means to reflect current cognitive function in SSVD patients. Furthermore, objective brain age-related MRI features in SSVD can be used as an effective aid to distinguish MCI patients from SCI patients with neurobiological interpretability.

## Data availability statement

The original contributions presented in this study are included in the article/Supplementary material, further inquiries can be directed to the corresponding authors.

## Ethics statement

The studies involving human participants were reviewed and approved by the Ethics Committee of The Affiliated Wuxi People’s Hospital of Nanjing Medical University. The patients/participants provided their written informed consent to participate in this study.

## Author contributions

YS, FW, and XF designed the study. YS drafted the manuscript. HM, QG, SZ, LM, and XZ collected the image data. GX, LL, ZW, WJ, PH, and KC recruited the participants, completed the neuropsychological assessments, and collected the blood samples. YS, HM, and QG analyzed the data. YY, JS, and XM contributed to the discussion. FW and XF contributed to the discussion and revised the manuscript. All authors contributed to the article and approved the submitted version.

## Footnotes

1
http://www.mlnl.cs.ucl.ac.uk/pronto/

2
https://sample-size.net/
