# Supplementary material for: Potential of brain age in identifying early cognitive impairment in subcortical small-vessel disease patients
Source: Front Aging Neurosci. 2022 Sep 1;14:973054. doi: 10.3389/fnagi.2022.973054 (PMC9475066; doi:10.3389/fnagi.2022.973054)
Supplement: Supplementary file 1 [file Data_Sheet_1.docx]

## Supplementary Materials

### Image acquisition and data preprocessing

A 3.0T MR scanner (MAGNETOM Prisma, Siemens Healthcare, Germany) is used in the present study. For each subject, the data of high-resolution T1-weighted images (T1WI) and resting-state functional MRI (rs-fMRI) was collected. None of subjects had excessive motion artifacts (≥ 2 mm translational or ≥ 2° rotational movements) or incomplete image coverage.

The scanning parameters were as follows: Protocol name = EPI; Repetition time = 1500 ms; Echo time = 31 ms; Flip angle = 70°; Field of view = 211 mm × 211 mm; Matrix = 88 × 88; Thickness (gap) = 2.4 mm (0); Slice number = 60.

### Grey matter density analysis

The statistical parametric mapping 8 (SPM8) toolbox (<http://www.fil.ion.ucl.ac.uk/spm/>) was used for the analysis of brain structural MRI data. The Diffeomorphic Anatomical Registration using Exponentiated Lie algebra (DARTEL) technique was used to generate a study-specific template. All images were spatially normalized using combinations of affine linear transformation and non-linear registration to the standard Montreal Neurological Institute (MNI) template and segmented into grey matter (GM), white matter (WM) and cerebrospinal fluid (CSF). Segmented GM images were modulated by multiplying the transformed gray matter density maps with the non-linear components of Jacobian determinants, which resulted in the normalized gray matter volume (GMV) maps representing the local native-space gray matter volume after correcting the confounding effect of variance induced by individual whole-brain size. The modulated GMV were resampled to 2-mm cubic voxel resolution and smoothed with a 3D Gaussian kernel (6 mm full-width half-maximum).

### Rs-fMRI data analysis

Rs-fMRI data were preprocessed using SPM8 and the Data Processing Assistant for Resting-State fMRI (DPARSF, <http://www.restfmri.net/forum/dparsf>) ([Chao-Gan and Yu-Feng, 2010](#_ENREF_2)). The initial 10 functional volumes were discarded for scanner stabilization and participant adaption. The remaining images were corrected for timing differences and motion effects. The individual structural images (T1WI) were co-registered to mean functional images after motion correction using linear transformation. Motion corrected functional volumes were spatially normalized to the Montreal Neurological Institute space using DARTEL toolbox ([Ashburner, 2007](#_ENREF_1)) and resampled to a voxel size of 3 mm × 3 mm × 3 mm. To further reduce the effects of confounding factors, Friston 24 motion parameters ([Friston et al., 1996](#_ENREF_5)), white matter signal, and cerebrospinal fluid signal were removed from the data via linear regression. Then, smoothing with a 6-mm full-width at half-maximum kernel and linear detrending were performed.

#### Amplitude of low-frequency fluctuation (ALFF) and fractional amplitude of low-frequency fluctuation (fALFF) analyses

DPARSF software was used for the present analyses ([Chao-Gan and Yu-Feng, 2010](#_ENREF_2)). Briefly, for each voxel, the time series was transformed to the frequency domain using Fast Fourier Transform and the power spectrum was acquired. Next, the square root was computed at each frequency of the power spectrum and average square root was obtained across 0.01 - 0.08 Hz at each voxel.

**For ALFF:** The averaged square root was taken as the ALFF. The ALFF of each voxel was divided by the global mean ALFF value for each participant, which provided a ‘mALFF’ spatial maps for each participant.

**For fALFF:** The sum of amplitude across 0.01-0.08 Hz was divided across the whole frequency range. The fALFF of each voxel was divided by the global average fALFF within a brain mask for standardization purpose, which provided a ‘mfALFF’ spatial maps for each participant.

# Multivariate relevance vector regression (RVR) analysis

RVR method is a sparse kernel learning multivariate regression method formulated using a fully probabilistic Bayesian framework, where a zero-mean Gaussian prior is cited over the model weights, and each weight is corresponding to a hyper-parameter ([Tipping, 2001](#_ENREF_7)). The training data are used to iteratively evaluate the most probable values for these hyper-parameters, and those training vectors associated with non-zero weights are identified as ‘relevance’ vectors.

The leave-one-out cross-validation (LOOCV) was performed to evaluate the out-of-sample prediction performance in the model ([Feng et al., 2019](#_ENREF_4)). *N-1* samples (*N* is the number of samples) were used as the training set, with the remaining samples used as the testing set. In the training procedure, each feature was linearly scaled to a range of zero to one across the training set, and then a RVR prediction model was constructed. In the testing procedure, each testing sample’s feature vector was scaled using the scaling parameter acquired during the training procedure. Subsequently, the RVR prediction model was used to predict brain age of each sample in the testing set ([Cui and Gong, 2018](#_ENREF_3)). The training and testing procedures were repeated N times and each sample was used once as the testing sample.

The Pearson correlation coefficient (R) and mean absolute error (MAE) between actual age and predicted brain age were used to assess the prediction performance. The permutation test was used to determine whether the obtained results were significantly better than those expected by chance. We permuted age across training samples without replacement 1000 times, and each time re-applied the above LOOCV prediction procedure, resulting in a distribution of R and MAE. The number of times that the permuted value was greater (or less) than or equal to the true value was then divided by 1000 providing a p-value for R and MAE ([Feng et al., 2019](#_ENREF_4); [Zhu et al., 2019](#_ENREF_8); [Shi et al., 2020](#_ENREF_6)).

Furthermore, the absolute value of weight value of each feature was used to quantify its contribution to prediction ([Cui and Gong, 2018](#_ENREF_3); [Feng et al., 2019](#_ENREF_4); [Zhu et al., 2019](#_ENREF_8); [Shi et al., 2020](#_ENREF_6)). In the RVR model, most weight will be zero; remaining ones with non-zero weight were used to fit the model. The regression coefficients of all features were determined as the weighted sum of the feature vector of the non-zero weighted samples. A larger absolute value of the weight of the corresponding feature indicated a greater contribution to prediction, in the context of all other features. The feature was retained if the absolute value of its weight was in the top 10% across all features. This threshold can eliminate noise components to some extent, thus enabling a better visualization of the most predictive features.

### Sample Size Calculation

The sample size calculation was performed using an online sample size calculators (<https://sample-size.net/>). We performed the sample size calculation (α=0.05, β=0.2) to confirm whether the sample size in SCI and MCI groups was acceptable based on the significantly different cognitive assessments and plasma oxidative stress biomarkers. The results of sample size calculation were displayed in Supplementary Table 2.

Therefore, the present sample size (SCI group: N = 24 and MCI group: N = 27) can provide an acceptable power (> 80%), and the sample size using in the present study can meet the minimum sample requirement based on results of the sample size calculation.

###
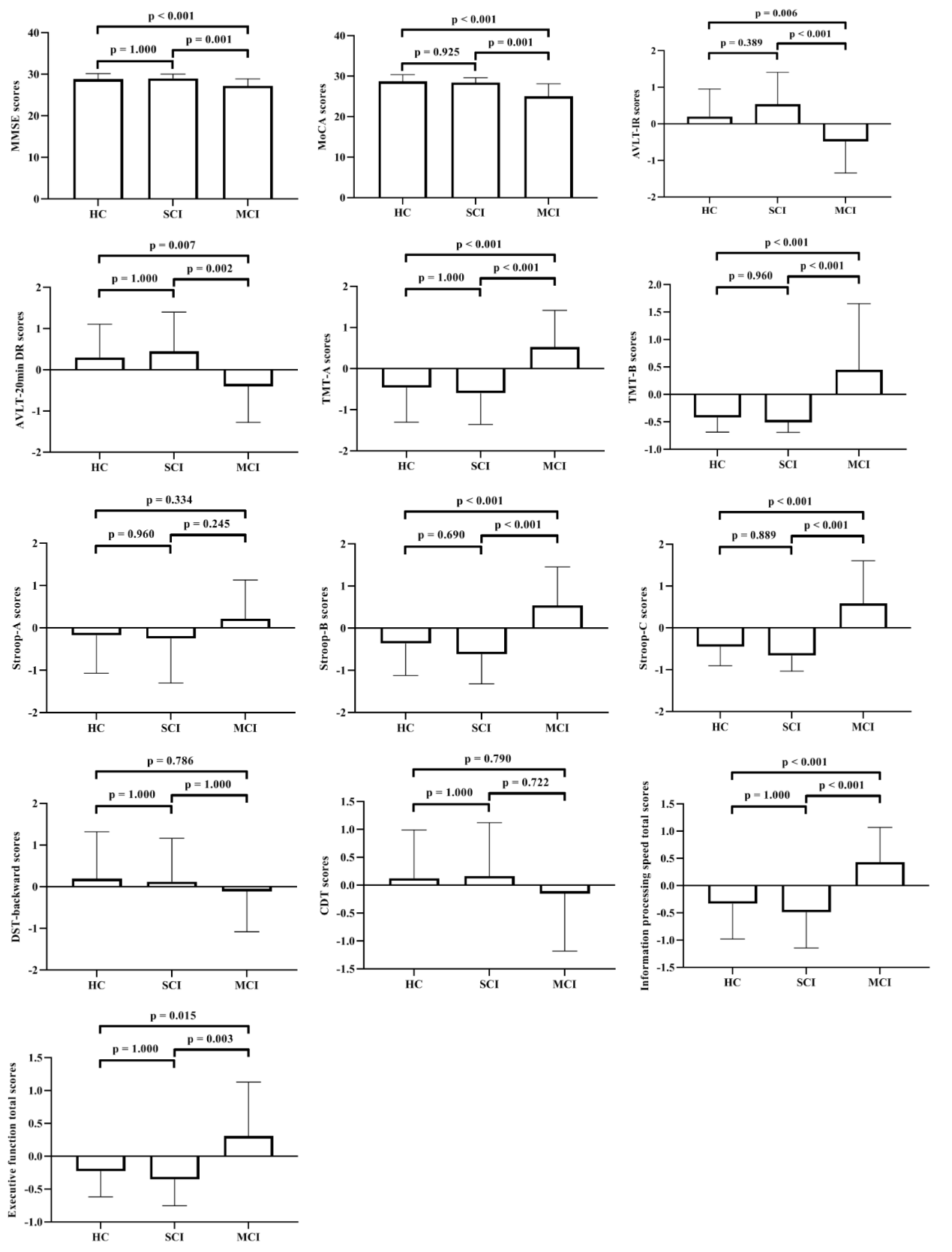


### Supplementary Figure 1. Difference analyses of cognitive assessments among HC, SCI, and MCI groups.

### Supplementary Table 1. Information of 246 brain regions extracted by the Brainnetome Atlas

| **ROI** | **MNI (X,Y,Z)** | **label** | **ROI** | **MNI (X,Y,Z)** |  | **label** | **ROI** | **MNI (X,Y,Z)** | **label** |
| --- | --- | --- | --- | --- | --- | --- | --- | --- | --- |
| 1 | -4,22,49 | SFG_L_7_1 | 89 | -46,-31,-23 |  | ITG_L_7_1 | 177 | -3,12,20 | CG_L_7_2 |
| 2 | 8,23,50 | SFG_R_7_1 | 90 | 45,-20,-32 |  | ITG_R_7_1 | 178 | 5,22,6 | CG_R_7_2 |
| 3 | -17,30,48 | SFG_L_7_2 | 91 | -51,-58,-8 |  | ITG_L_7_2 | 179 | -5,35,14 | CG_L_7_3 |
| 4 | 23,32,45 | SFG_R_7_2 | 92 | 53,-56,-16 |  | ITG_R_7_2 | 180 | 6,31,22 | CG_R_7_3 |
| 5 | -10,53,32 | SFG_L_7_3 | 93 | -45,-8,-39 |  | ITG_L_7_3 | 181 | -8,-45,13 | CG_L_7_4 |
| 6 | 14,53,32 | SFG_R_7_3 | 94 | 39,-3,-41 |  | ITG_R_7_3 | 182 | 9,-43,13 | CG_R_7_4 |
| 7 | -18,8,61 | SFG_L_7_4 | 95 | -56,-19,-27 |  | ITG_L_7_4 | 183 | -4,11,34 | CG_L_7_5 |
| 8 | 22,11,59 | SFG_R_7_4 | 96 | 54,-15,-33 |  | ITG_R_7_4 | 184 | 5,11,35 | CG_R_7_5 |
| 9 | -5,2,54 | SFG_L_7_5 | 97 | -56,-61,0 |  | ITG_L_7_5 | 185 | -6,-17,41 | CG_L_7_6 |
| 10 | 8,4,57 | SFG_R_7_5 | 98 | 53,-59,-4 |  | ITG_R_7_5 | 186 | 8,-15,41 | CG_R_7_6 |
| 11 | -4,40,32 | SFG_L_7_6 | 99 | -60,-43,-12 |  | ITG_L_7_6 | 187 | -4,38,-9 | CG_L_7_7 |
| 12 | 7,42,29 | SFG_R_7_6 | 100 | 60,-43,-14 |  | ITG_R_7_6 | 188 | 6,41,0 | CG_R_7_7 |
| 13 | -7,56,7 | SFG_L_7_7 | 101 | -55,-35,-24 |  | ITG_L_7_7 | 189 | -12,-83,-3 | MVOcC_L_5_1 |
| 14 | 8,58,5 | SFG_R_7_7 | 102 | 54,-35,-25 |  | ITG_R_7_7 | 190 | 9,-86,-1 | MVOcC_R_5_1 |
| 15 | -26,46,24 | MFG_L_7_1 | 103 | -35,-25,-25 |  | FuG_L_3_1 | 191 | -6,-79,18 | MVOcC_L_5_2 |
| 16 | 31,41,28 | MFG_R_7_1 | 104 | 34,-24,-29 |  | FuG_R_3_1 | 192 | 7,-74,18 | MVOcC_R_5_2 |
| 17 | -41,18,33 | MFG_L_7_2 | 105 | -32,-66,-8 |  | FuG_L_3_2 | 193 | -6,-93,10 | MVOcC_L_5_3 |
| 18 | 43,16,33 | MFG_R_7_2 | 106 | 30,-64,-9 |  | FuG_R_3_2 | 194 | 8,-88,21 | MVOcC_R_5_3 |
| 19 | -27,57,4 | MFG_L_7_3 | 107 | -42,-52,-13 |  | FuG_L_3_3 | 195 | -17,-61,-1 | MVOcC_L_5_4 |
| 20 | 29,56,8 | MFG_R_7_3 | 108 | 42,-52,-15 |  | FuG_R_3_3 | 196 | 17,-61,-2 | MVOcC_R_5_4 |
| 21 | -40,43,10 | MFG_L_7_4 | 109 | -28,-11,-35 |  | PhG_L_6_1 | 197 | -13,-66,18 | MVOcC_L_5_5 |
| 22 | 43,44,5 | MFG_R_7_4 | 110 | 26,-13,-35 |  | PhG_R_6_1 | 198 | 15,-62,17 | MVOcC_R_5_5 |
| 23 | -32,29,41 | MFG_L_7_5 | 111 | -26,-28,-25 |  | PhG_L_6_2 | 199 | -30,-88,20 | LOcC_L_4_1 |
| 24 | 43,31,33 | MFG_R_7_5 | 112 | 26,-27,-27 |  | PhG_R_6_2 | 200 | 34,-83,19 | LOcC_R_4_1 |
| 25 | -31,11,53 | MFG_L_7_6 | 113 | -29,-33,-16 |  | PhG_L_6_3 | 201 | -46,-73,11 | LOcC_L_4_2 |
| 26 | 35,14,50 | MFG_R_7_6 | 114 | 29,-32,-18 |  | PhG_R_6_3 | 202 | 47,-70,5 | LOcC_R_4_2 |
| 27 | -25,59,-12 | MFG_L_7_7 | 115 | -20,-16,-30 |  | PhG_L_6_4 | 203 | -18,-99,12 | LOcC_L_4_3 |
| 28 | 25,58,-11 | MFG_R_7_7 | 116 | 18,-14,-30 |  | PhG_R_6_4 | 204 | 22,-96,13 | LOcC_R_4_3 |
| 29 | -45,16,21 | IFG_L_6_1 | 117 | -24,-2,-33 |  | PhG_L_6_5 | 205 | -31,-89,-4 | LOcC_L_4_4 |
| 30 | 46,18,20 | IFG_R_6_1 | 118 | 21,-4,-38 |  | PhG_R_6_5 | 206 | 31,-86,-4 | LOcC_R_4_4 |
| 31 | -47,34,9 | IFG_L_6_2 | 119 | -18,-41,-7 |  | PhG_L_6_6 | 207 | -11,-83,39 | LOcC_L_2_1 |
| 32 | 49,35,5 | IFG_R_6_2 | 120 | 18,-38,-9 |  | PhG_R_6_6 | 208 | 16,-80,42 | LOcC_R_2_1 |
| 33 | -53,24,7 | IFG_L_6_3 | 121 | -54,-39,8 |  | pSTS_L_2_1 | 209 | -22,-72,43 | LOcC_L_2_2 |
| 34 | 54,23,6 | IFG_R_6_3 | 122 | 52,-37,5 |  | pSTS_R_2_1 | 210 | 29,-69,42 | LOcC_R_2_2 |
| 35 | -49,36,-8 | IFG_L_6_4 | 123 | -53,-49,15 |  | pSTS_L_2_2 | 211 | -20,-6,-21 | Amyg_L_2_1 |
| 36 | 51,35,-8 | IFG_R_6_4 | 124 | 57,-39,14 |  | pSTS_R_2_2 | 212 | 18,-5,-22 | Amyg_R_2_1 |
| 37 | -39,21,0 | IFG_L_6_5 | 125 | -15,-52,68 |  | SPL_L_5_1 | 213 | -28,-7,-21 | Amyg_L_2_2 |
| 38 | 42,22,-2 | IFG_R_6_5 | 126 | 20,-49,68 |  | SPL_R_5_1 | 214 | 27,-6,-22 | Amyg_R_2_2 |
| 39 | -52,14,4 | IFG_L_6_6 | 127 | -16,-63,57 |  | SPL_L_5_2 | 215 | -22,-16,-20 | Hipp_L_2_1 |
| 40 | 54,14,6 | IFG_R_6_6 | 128 | 19,-62,59 |  | SPL_R_5_2 | 216 | 23,-16,-20 | Hipp_R_2_1 |
| 41 | -7,51,-15 | OrG_L_6_1 | 129 | -33,-40,54 |  | SPL_L_5_3 | 217 | -28,-30,-8 | Hipp_L_2_2 |
| 42 | 6,46,-15 | OrG_R_6_1 | 130 | 37,-35,54 |  | SPL_R_5_3 | 218 | 28,-30,-9 | Hipp_R_2_2 |
| 43 | -36,30,-21 | OrG_L_6_2 | 131 | -22,-39,68 |  | SPL_L_5_4 | 219 | -13,14,-4 | BG_L_6_1 |
| 44 | 40,34,-21 | OrG_R_6_2 | 132 | 25,-35,67 |  | SPL_R_5_4 | 220 | 14,13,-6 | BG_R_6_1 |
| 45 | -23,34,-23 | OrG_L_6_3 | 133 | -26,-51,60 |  | SPL_L_5_5 | 221 | -22,-2,2 | BG_L_6_2 |
| 46 | 24,31,-23 | OrG_R_6_3 | 134 | 32,-48,57 |  | SPL_R_5_5 | 222 | 22,-2,1 | BG_R_6_2 |
| 47 | -6,48,-26 | OrG_L_6_4 | 135 | -34,-76,37 |  | IPL_L_6_1 | 223 | -17,2,-11 | BG_L_6_3 |
| 48 | 6,51,-25 | OrG_R_6_4 | 136 | 45,-68,25 |  | IPL_R_6_1 | 224 | 14,7,-12 | BG_R_6_3 |
| 49 | -11,15,-22 | OrG_L_6_5 | 137 | -37,-54,53 |  | IPL_L_6_2 | 225 | -23,6,-6 | BG_L_6_4 |
| 50 | 9,16,-23 | OrG_R_6_5 | 138 | 41,-59,48 |  | IPL_R_6_2 | 226 | 22,7,-5 | BG_R_6_4 |
| 51 | -40,30,-14 | OrG_L_6_6 | 139 | -50,-28,45 |  | IPL_L_6_3 | 227 | -14,3,14 | BG_L_6_5 |
| 52 | 42,29,-15 | OrG_R_6_6 | 140 | 49,-29,45 |  | IPL_R_6_3 | 228 | 14,7,11 | BG_R_6_5 |
| 53 | -49,-2,38 | PrG_L_6_1 | 141 | -55,-45,42 |  | IPL_L_6_4 | 229 | -28,-5,1 | BG_L_6_6 |
| 54 | 54,0,31 | PrG_R_6_1 | 142 | 57,-39,40 |  | IPL_R_6_4 | 230 | 29,-4,-1 | BG_R_6_6 |
| 55 | -31,-1,56 | PrG_L_6_2 | 143 | -46,-61,33 |  | IPL_L_6_5 | 231 | -7,-12,5 | Tha_L_8_1 |
| 56 | 34,-1,54 | PrG_R_6_2 | 144 | 53,-51,28 |  | IPL_R_6_5 | 232 | 7,-11,5 | Tha_R_8_1 |
| 57 | -26,-17,62 | PrG_L_6_3 | 145 | -54,-28,26 |  | IPL_L_6_6 | 233 | -19,-13,3 | Tha_L_8_2 |
| 58 | 36,-12,57 | PrG_R_6_3 | 146 | 55,-23,25 |  | IPL_R_6_6 | 234 | 13,-14,1 | Tha_R_8_2 |
| 59 | -11,-14,69 | PrG_L_6_4 | 147 | -4,-56,56 |  | PCun_L_4_1 | 235 | -18,-22,5 | Tha_L_8_3 |
| 60 | 17,-15,67 | PrG_R_6_4 | 148 | 7,-58,56 |  | PCun_R_4_1 | 236 | 17,-22,3 | Tha_R_8_3 |
| 61 | -52,2,7 | PrG_L_6_5 | 149 | -7,-40,60 |  | PCun_L_4_2 | 237 | -7,-15,6 | Tha_L_8_4 |
| 62 | 54,3,5 | PrG_R_6_5 | 150 | 8,-40,61 |  | PCun_R_4_2 | 238 | 3,-13,5 | Tha_R_8_4 |
| 63 | -48,9,30 | PrG_L_6_6 | 151 | -13,-62,30 |  | PCun_L_4_3 | 239 | -17,-24,7 | Tha_L_8_5 |
| 64 | 52,10,26 | PrG_R_6_6 | 152 | 15,-60,31 |  | PCun_R_4_3 | 240 | 15,-24,6 | Tha_R_8_5 |
| 65 | -8,-29,57 | PCL_L_2_1 | 153 | -6,-50,38 |  | PCun_L_4_4 | 241 | -15,-27,6 | Tha_L_8_6 |
| 66 | 10,-29,57 | PCL_R_2_1 | 154 | 7,-49,38 |  | PCun_R_4_4 | 242 | 13,-27,8 | Tha_R_8_6 |
| 67 | -3,-15,60 | PCL_L_2_2 | 155 | -49,-10,44 |  | PoG_L_4_1 | 243 | -11,-18,13 | Tha_L_8_7 |
| 68 | 5,-14,60 | PCL_R_2_2 | 156 | 50,-10,42 |  | PoG_R_4_1 | 244 | 10,-13,14 | Tha_R_8_7 |
| 69 | -33,10,-35 | STG_L_6_1 | 157 | -56,-12,17 |  | PoG_L_4_2 | 245 | -11,-14,2 | Tha_L_8_8 |
| 70 | 31,10,-36 | STG_R_6_1 | 158 | 56,-9,13 |  | PoG_R_4_2 | 246 | 13,-17,6 | Tha_R_8_8 |
| 71 | -53,-30,15 | STG_L_6_2 | 159 | -45,-23,52 |  | PoG_L_4_3 |  |  |  |
| 72 | 54,-23,10 | STG_R_6_2 | 160 | 48,-19,48 |  | PoG_R_4_3 |  |  |  |
| 73 | -50,-10,2 | STG_L_6_3 | 161 | -22,-26,67 |  | PoG_L_4_4 |  |  |  |
| 74 | 50,-6,-4 | STG_R_6_3 | 162 | 25,-24,66 |  | PoG_R_4_4 |  |  |  |
| 75 | -64,-33,13 | STG_L_6_4 | 163 | -36,-19,11 |  | INS_L_6_1 |  |  |  |
| 76 | 66,-22,6 | STG_R_6_4 | 164 | 37,-18,7 |  | INS_R_6_1 |  |  |  |
| 77 | -45,8,-21 | STG_L_6_5 | 165 | -32,12,-16 |  | INS_L_6_2 |  |  |  |
| 78 | 46,9,-24 | STG_R_6_5 | 166 | 32,12,-17 |  | INS_R_6_2 |  |  |  |
| 79 | -56,-2,-10 | STG_L_6_6 | 167 | -34,17,-2 |  | INS_L_6_3 |  |  |  |
| 80 | 55,-14,-8 | STG_R_6_6 | 168 | 36,18,-4 |  | INS_R_6_3 |  |  |  |
| 81 | -65,-32,-10 | MTG_L_4_1 | 169 | -39,-5,-10 |  | INS_L_6_4 |  |  |  |
| 82 | 65,-31,-12 | MTG_R_4_1 | 170 | 39,-4,-11 |  | INS_R_6_4 |  |  |  |
| 83 | -54,-2,-30 | MTG_L_4_2 | 171 | -38,-7,8 |  | INS_L_6_5 |  |  |  |
| 84 | 52,0,-33 | MTG_R_4_2 | 172 | 39,-7,6 |  | INS_R_6_5 |  |  |  |
| 85 | -59,-57,10 | MTG_L_4_3 | 173 | -37,5,2 |  | INS_L_6_6 |  |  |  |
| 86 | 60,-53,6 | MTG_R_4_3 | 174 | 39,5,2 |  | INS_R_6_6 |  |  |  |
| 87 | -58,-20,-8 | MTG_L_4_4 | 175 | -3,-35,34 |  | CG_L_7_1 |  |  |  |
| 88 | 59,-19,-11 | MTG_R_4_4 | 176 | 5,-32,34 |  | CG_R_7_1 |  |  |  |

#

### Supplementary Table 2. Sample size calculation for SCI and MCI groups

|  | **SCI group (N)** | **MCI (N)** | **Power** |
| --- | --- | --- | --- |
| MMSE score | 11 | 11 | 0.804 |
| MoCA score | 9 | 9 | 0.808 |
| AVLT-IR (Z score) | 13 | 13 | 0.822 |
| AVLT-20min DR (Z score) | 20 | 20 | 0.816 |
| TMT-A (Z score) | 10 | 10 | 0.816 |
| TMT-B (Z score) | 14 | 14 | 0.813 |
| Stroop-B (Z score) | 9 | 9 | 0.812 |
| Stroop-C (Z score) | 7 | 7 | 0.838 |
| Information processing speed | 9 | 9 | 0.805 |
| Executive function | 16 | 16 | 0.826 |
| Plasma T-AOC (U/ml) | 22 | 22 | 0.810 |

**NOTE.** The sample size calculation (α=0.05, β=0.2) to confirm whether the sample size of SCI and MCI groups (24 SCI and 27 MCI) was acceptable based on the significantly different indices of Table 1.

### Supplementary Table 3. Construction of RVR models with MRI features for predicting the brain age of 35 HCs

| **MRI features** | **Prediction models** |
| --- | --- |
| ALFF features | R = 0.446, MAE = 5.944 |
| fALFF features | R = -0.116, MAE = 8.381 |
| ALFF + GMV features | R = 0.489, MAE = 5.278 |
| fALFF + GMV features | R = 0.112, MAE = 7.560 |

### Supplementary Table 4. Association of age with cognitive function and plasma antioxidant index levels in MCI patients.

|  | chronological age^*^ | estimated age^*^ | estimated age^#^ | brain age gap^*^ | brain age gap^#^ |
| --- | --- | --- | --- | --- | --- |
| MMSE | r = 0.358; p = 0.067 | r = -0.188; p = 0.348 | r = -0.286; p = 0.186 | **r = -0.558; p = 0.002** | **r = -0.449; p = 0.032** |
| MoCA | r = 0.315; p = 0.061 | r = -0.139; p = 0.489 | r = -0.247; p = 0.256 | **r = -0.475; p = 0.012** | r = -0.277; p = 0.200 |
| AVLT-IR | r = 0.090; p = 0.654 | **r = -0.428; p = 0.026** | **r = -0.526; p = 0.010** | **r = -0.500; p = 0.008** | **r = -0.632; p = 0.001** |
| AVLT-20min DR | r = 0.282; p = 0.154 | r = -0.199; p = 0.319 | r = -0.298; p = 0.167 | **r = -0.456; p = 0.017** | r = -0.389; p = 0.067 |
| TMT-A | r = 0.084; p = 0.679 | r = 0.234; p = 0.240 | r = 0.186; p = 0.395 | r = 0.176; p = 0.381 | r = 0.280; p = 0.196 |
| TMT-B | r = 0.024; p = 0.907 | **r = 0.573; p = 0.002** | **r = 0.622; p = 0.002** | **r = 0.547; p = 0.003** | **r = 0.777; p < 0.001** |
| Stroop-A | r = -0.056 ; p = 0.782 | r = 0.236; p = 0.237 | r = 0.320; p = 0.137 | r = 0.302; p = 0.126 | r = 0.347; p = 0.104 |
| Stroop-B | r = -0.300 ; p = 0.129 | r = 0.080; p = 0.692 | r = 0.198; p = 0.364 | r = 0.354; p = 0.070 | r = 0.263; p = 0.225 |
| Stroop-C | r = -0.065; p = 0.748 | **r = 0.525; p = 0.005** | **r = 0.589; p = 0.003** | **r = 0.570; p = 0.002** | **r = 0.701; p < 0.001** |
| DST-backward | r = -0.276; p = 0.164 | r = 0.247; p = 0.215 | r = 0.381; p = 0.073 | r = 0.349; p = 0.103 | r = 0.358; p = 0.098 |
| CDT | r = -0.192; p = 0.338 | r = 0.079; p = 0.694 | r = 0.169; p = 0.440 | r = 0.187; p = 0.351 | r = 0.111; p = 0.613 |
| Information processing speed | r = -0.130; p = 0.519 | r = 0.258; p = 0.193 | r = 0.324; p = 0.131 | **r = 0.392; p = 0.043** | **r = 0.407; p = 0.050** |
| Executive function | r = -0.124; p = 0.539 | **r = 0.594; p = 0.001** | **r = 0.679; p < 0.001** | **r = 0.668; p < 0.001** | **r = 0.785; p < 0.001** |

**NOTE:** (1) Z scores of other assessments were used for the present analysis except for the use of raw scores of the MMSE and MoCA. (2) The information processing speed total scores were calculated using the TMT-A, Stroop-A, and Stroop-B scales (Z scores) scores, while the executive function total scores were calculated using the TMT-B, Stroop-C, and DST-backward scale (Z scores) scores. (3) Brain age gap = (estimated age - chronological age).

**Abbreviations:** MCI, mild cognitive impairment; MMSE, Mini-mental State Examination; MoCA, Montreal Cognitive Assessment; AVLT-IR, Auditory Verbal Learning Test-immediate recall; AVLT-20min DR, Auditory Verbal Learning Test-20-minute delayed recall; TMT-A, Trail Making Test-A; Stroop, Stroop Color and Word Test; TMT-B, Trail Making Test-B; DST, Digit Span Test; CDT, Clock Drawing Test.

^*^ P-values were obtained by Pearson correlation test.

^#^ P-values were obtained by Partial correlation test (adjusting chronological age, sex, years of education, and NIHSS score).

### Supplementary Table 5. Association of age with cognitive function and plasma antioxidant index levels in SCI patients.

|  | chronological age^*^ | estimated age^*^ | estimated age^#^ | brain age gap^*^ | brain age gap^#^ |
| --- | --- | --- | --- | --- | --- |
| MMSE | r = 0.232; p = 0.275 | r = -0.044; p = 0.838 | r = 0.028; p = 0.905 | r = -0.172; p = 0.423 | r = -0.147; p = 0.526 |
| MoCA | r = 0.059; p = 0.785 | **r = -0.380; p = 0.067** | r = -0.288; p = 0.206 | **r = -0.370; p = 0.044** | r = -0.286; p = 0.209 |
| AVLT-IR | r = -0.115; p = 0.592 | r = 0.114; p = 0.597 | r = 0.245; p = 0.285 | r = 0.164; p = 0.445 | r = 0.260; p = 0.255 |
| AVLT-20min DR | r = -0.015; p = 0.945 | r = 0.007; p = 0.973 | r = 0.154; p = 0.506 | r = 0.015; p = 0.946 | r = 0.109; p = 0.640 |
| TMT-A | **r = 0.545; p = 0.006** | r = 0.304; p = 0.149 | r = 0.047; p = 0.841 | r = -0.054 ; p = 0.803 | r = -0.273; p = 0.231 |
| TMT-B | r = 0.196; p = 0.359 | **r = 0.391; p = 0.043** | r = 0.031; p = 0.892 | r = 0.188; p = 0.380 | r = -0.030; p = 0.899 |
| Stroop-A | r = 0.036; p = 0.869 | r = 0.035; p = 0.869 | r = 0.031; p = 0.894 | r = 0.010; p = 0.964 | r = -0.028 ; p = 0.904 |
| Stroop-B | r = 0.222; p = 0.298 | r = -0.321 ; p = 0.126 | r = -0.296 ; p = 0.193 | **r = -0.403; p = 0.050** | **r = -0.447; p = 0.042** |
| Stroop-C | r = 0.275; p = 0.193 | r = -0.206 ; p = 0.333 | r = -0.223; p = 0.332 | **r = -0.521; p = 0.009** | **r = -0.573; p = 0.007** |
| DST-backward | r = 0.123; p = 0.567 | r = -0.098; p = 0.650 | r = -0.034; p = 0.882 | r = -0.155; p = 0.471 | r = -0.101; p = 0.663 |
| CDT | r = 0.185; p = 0.387 | **r = -0.406; p = 0.049** | r = -0.332; p = 0.142 | r = -0.455; p = 0.026 | r = -0.419; p = 0.059 |
| Information processing speed | r = 0.309; p = 0.141 | r = 0.022; p = 0.918 | r = -0.072; p = 0.756 | r = -0.159 ; p = 0.457 | r = -0.285; p = 0.210 |
| Executive function | r = 0.320; p = 0.128 | r = -0.095; p = 0.658 | r = -0.035; p = 0.880 | r = -0.266; p = 0.209 | r = -0.268; p = 0.240 |

**NOTE:** (1) Z scores of other assessments were used for the present analysis except for the use of raw scores of the MMSE and MoCA. (2) The information processing speed total scores were calculated using the TMT-A, Stroop-A, and Stroop-B scales (Z scores) scores, while the executive function total scores were calculated using the TMT-B, Stroop-C, and DST-backward scale (Z scores) scores. (3) Brain age gap = (estimated age - chronological age).

**Abbreviations:** SCI, subjective cognitive impairment; MMSE, Mini-mental State Examination; MoCA, Montreal Cognitive Assessment; AVLT-IR, Auditory Verbal Learning Test-immediate recall; AVLT-20min DR, Auditory Verbal Learning Test-20-minute delayed recall; TMT-A, Trail Making Test-A; Stroop, Stroop Color and Word Test; TMT-B, Trail Making Test-B; DST, Digit Span Test; CDT, Clock Drawing Test.

^*^ P-values were obtained by Pearson correlation test.

^#^ P-values were obtained by Partial correlation test (adjusting chronological age, sex, years of education, and NIHSS score)

### Supplementary Table 6. Association of age with cognitive function and plasma antioxidant index levels in HC participants.

|  | chronological age^*^ | estimated age^*^ | estimated age^#^ | brain age gap^*^ | brain age gap^#^ |
| --- | --- | --- | --- | --- | --- |
| MMSE | r = -0.320; p = 0.061 | r = -0.242; p = 0.162 | r = -0.195; p = 0.277 | r = 0.132; p = 0.448 | r = 0.064; p = 0.724 |
| MoCA | r = -0.267; p = 0.122 | r = -0.166; p = 0.339 | r = -0.187; p = 0.298 | r = 0.156; p = 0.371 | r = 0.077; p = 0.671 |
| AVLT-IR | **r = -0.349; p = 0.040** | r = -0.182; p = 0.295 | r = -0.068; p = 0.707 | r = 0.252; p = 0.145 | r = 0.207; p = 0.249 |
| AVLT-20min DR | r = -0.443; p = 0.008 | r = -0.228; p = 0.188 | r = -0.030; p = 0.870 | r = 0.323; p = 0.059 | r = 0.258; p = 0.147 |
| TMT-A | **r = 0.537; p = 0.001** | **r = 0.555; p = 0.001** | **r = 0.531; p = 0.001** | r = -0.027; p = 0.879 | r = 0.066; p = 0.714 |
| TMT-B | r = 0.359; p = 0.034 | r = 0.314; p = 0.073 | r = 0.320; p = 0.069 | r = 0.039; p = 0.825 | r = 0.088; p = 0.627 |
| Stroop-A | **r = 0.534; p = 0.001** | **r = 0.520; p = 0.001** | **r = 0.419; p = 0.015** | r = -0.067 ; p = 0.703 | r = 0.061; p = 0.737 |
| Stroop-B | r = 0.241; p = 0.163 | r = 0.194; p = 0.264 | r = 0.056; p = 0.758 | r = -0.084; p = 0.631 | r = -0.087; p = 0.631 |
| Stroop-C | **r = 0.459; p = 0.006** | **r = 0.420; p = 0.012** | r = 0.314; p = 0.075 | r = -0.093; p = 0.596 | r = 0.001; p = 0.996 |
| DST-backward | **r = -0.363; p = 0.032** | r = -0.306; p = 0.074 | r = -0.274; p = 0.123 | r = 0.109; p = 0.532 | r = -0.033; p = 0.854 |
| CDT | r = -0.326; p = 0.056 | r = -0.305; p = 0.074 | r = -0.283; p = 0.111 | r = 0.057; p = 0.747 | r = 0.043; p = 0.812 |
| Information processing speed | **r = 0.573; p < 0.001** | **r = 0.556; p = 0.001** | **r = 0.463; p = 0.007** | r = -0.075; p = 0.667 | r = 0.025; p = 0.891 |
| Executive function | r = -0.089; p = 0.609 | r = -0.037; p = 0.833 | r = -0.079; p = 0.660 | r = 0.077; p = 0.659 | r = 0.050; p = 0.781 |

**NOTE:** (1) Z scores of other assessments were used for the present analysis except for the use of raw scores of the MMSE and MoCA. (2) The information processing speed total scores were calculated using the TMT-A, Stroop-A, and Stroop-B scales (Z scores) scores, while the executive function total scores were calculated using the TMT-B, Stroop-C, and DST-backward scale (Z scores) scores. (3) Brain age gap = (estimated age - chronological age).

**Abbreviations:** HC, healthy control; MMSE, Mini-mental State Examination; MoCA, Montreal Cognitive Assessment; AVLT-IR, Auditory Verbal Learning Test-immediate recall; AVLT-20min DR, Auditory Verbal Learning Test-20-minute delayed recall; TMT-A, Trail Making Test-A; Stroop, Stroop Color and Word Test; TMT-B, Trail Making Test-B; DST, Digit Span Test; CDT, Clock Drawing Test.

^*^ P-values were obtained by Pearson correlation test.

^#^ P-values were obtained by Partial correlation test (adjusting chronological age, sex, years of education, and NIHSS score)

# Reference

Ashburner J (2007). A fast diffeomorphic image registration algorithm. Neuroimage, 38(1): 95-113

Chao-Gan Y, Yu-Feng Z (2010). DPARSF: A MATLAB Toolbox for "Pipeline" Data Analysis of Resting-State fMRI. Frontiers in systems neuroscience, 4: 13

Cui Z, Gong G (2018). The effect of machine learning regression algorithms and sample size on individualized behavioral prediction with functional connectivity features. Neuroimage, 178: 622-637

Feng C, Cui Z, Cheng D, Xu R, Gu R (2019). Individualized prediction of dispositional worry using white matter connectivity. Psychol Med, 49(12): 1999-2008

Friston K J, Williams S, Howard R, Frackowiak R S, Turner R (1996). Movement-related effects in fMRI time-series. Magnetic Resonance in Medicine, 35(3): 346-355

Shi Y, Wang Z, Chen P, Cheng P, Zhao K, Zhang H, Shu H, Gu L, Gao L, Wang Q, Zhang H, Xie C, Liu Y, Zhang Z (2020). Episodic Memory-Related Imaging Features as Valuable Biomarkers for the Diagnosis of Alzheimer's Disease: A Multicenter Study Based on Machine Learning. Biol Psychiatry Cogn Neurosci Neuroimaging,

Tipping M E (2001). Sparse Bayesian learning and the relevance vector machine. J Mach Learn Res, 1(3): 211-244

Zhu J, Zhu D M, Zhang C, Wang Y, Yang Y, Yu Y (2019). Quantitative prediction of individual cognitive flexibility using structural MRI. Brain Imaging Behav, 13(3): 781-788
